# Two-year follow-up of 4 months metformin treatment vs. placebo in ST-elevation myocardial infarction: data from the GIPS-III RCT

**DOI:** 10.1007/s00392-017-1140-z

**Published:** 2017-07-28

**Authors:** Minke H. T. Hartman, Jake K. B. Prins, Remco A. J. Schurer, Erik Lipsic, Chris P. H. Lexis, Anouk N. A. van der Horst-Schrivers, Dirk J. van Veldhuisen, Iwan C. C. van der Horst, Pim van der Harst

**Affiliations:** 1 Department of Cardiology, University of Groningen, University Medical Center Groningen, Hanzeplein 1, 9700 RB Groningen, The Netherlands; 2 Department of Endocrinology and Metabolism, University of Groningen, University Medical Center Groningen, Groningen, The Netherlands; 3 Department of Critical Care, University of Groningen, University Medical Center Groningen, Groningen, The Netherlands

**Keywords:** Acute myocardial infarction, Diabetes, Heart failure, Percutaneous coronary intervention, Metformin

## Abstract

**Objectives:**

Preclinical and clinical studies suggested cardioprotective effects of metformin treatment. In the GIPS-III trial, 4 months of metformin treatment did not improve left ventricular ejection fraction in patients presenting with ST-elevation myocardial infarction (STEMI). Here, we report the 2-year follow-up results.

**Methods:**

Between January 2011 and May 2013, 379 STEMI patients without diabetes undergoing primary percutaneous coronary intervention were randomized to a 4-month treatment with metformin (500 mg twice daily) (*N* = 191) or placebo (*N* = 188) in the University Medical Center Groningen. Two-year follow-up data was collected to determine its effect on predefined secondary endpoints: the incidence of major adverse cardiac events (MACE), its individual components, all-cause mortality, and new-onset diabetes.

**Results:**

For all 379 patients all-cause mortality data were available. For seven patients (2%) follow-up data on MACE was limited, ranging from 129 to 577 days. All others completed the 2-year follow-up visit. Incidence of MACE was 11 (5.8%) in metformin and 6 (3.2%) in placebo treated patients [hazard ratio (HR) 1.84, confidence interval (CI) 0.68–4.97, *P* = 0.22]. Three patients died in the metformin group and one in the placebo treatment group. Individual components of MACE were also comparable between both groups. New-onset diabetes mellitus was 34 (17.8%) in metformin and 32 (17.0%) in placebo treated patients (odds ratio 1.15, CI 0.66–1.98, *P* = 0.84). After multivariable adjustment the incidence of MACE was comparable between the treatment groups (HR 1.02, CI 0.10–10.78, *P* = 0.99).

**Conclusions:**

Four months metformin treatment initiated at the time of hospitalization in STEMI patients without diabetes did not exert beneficial long-term effects.

**Trial registration:**

clinicaltrials.gov Identifier: NCT01217307.

**Electronic supplementary material:**

The online version of this article (doi:10.1007/s00392-017-1140-z) contains supplementary material, which is available to authorized users.

## Introduction

The prognosis of patients presenting with ST-segment elevation myocardial infarction (STEMI) has substantially improved over the last decades. However, the development of heart failure remains an important cause of morbidity and mortality for STEMI patients and new strategies to reduce this risk are warranted [1].

The dimethylbiguanide metformin, used as first-line treatment in patients with type II diabetes mellitus (DM), has been suggested to exhibit cardioprotective effects in the setting of myocardial infarction (MI) in both preclinical and clinical studies irrespective of its glucose-lowering properties [2, 3]. Metformin treatment prior to reperfusion has been shown to reduce infarct size in several animal experimental studies as well as in observational studies in patients with DM [4]. Outside of the setting of MI, chronic metformin treatment in patients with type II DM was also associated with lower N-terminal pro-brain natriuretic peptide (NT pro-BNP) levels, further supporting a potential beneficial effect on the risk of heart failure [5]. Furthermore, a favorable effect on adverse remodeling has been observed in non-diabetic mice undergoing permanent coronary artery ligation, where metformin treatment during reperfusion improved left ventricular ejection fraction (LVEF) [2]. To test the hypothesis that metformin protects the heart against adverse cardiac remodeling after STEMI we designed and executed the glycometabolic intervention as adjunct to primary percutaneous coronary intervention in ST-segment elevation myocardial infarction (GIPS) III trial, a randomized, double blinded clinical trial. We previously published the primary findings of GIPS-III [6]. Four months of metformin treatment in patients without DM presenting with STEMI and undergoing primary percutaneous coronary intervention (PCI) had no improvement on LVEF. In addition, no significant effect was seen on NT pro-BNP levels after 4 months; in both groups the median NT pro-BNP was 167 ng/L (*P* = 0.66). Glucose regulation was also comparable at 4 months, despite metformin treatment [7].

Here, we report the long-term effects of 4 months of metformin treatment in STEMI patients without DM on predefined secondary endpoints, including major adverse cardiac events (MACE).

## Methods

The GIPS-III trial was a prospective, double-blind randomized clinical trial. The included patients were admitted via the STEMI protocol to the University Medical Center of Groningen. Inclusion criteria were: age of 18 years or older, STEMI diagnosis, and successful PCI with at least one ≥3 mm stent resulting in a subsequent thrombolysis in myocardial infarction (TIMI) flow grade of 2 or 3. Previous MI, diagnosis of DM, the need for coronary artery bypass grafting (CABG), severe renal impairment, and contra-indications for cardiac magnetic resonance imaging (CMR) were considered exclusion criteria. A detailed description of the study design, and the rationale of the GIPS-III trial as well as the primary results have been published [6, 8]. The trial was registered at clincialtrials.gov (NCT01217307).

The study procedures have been described in detail previously [6, 8]. In brief, standard laboratory assessment and physical examination were performed on admission followed by coronary angiography and PCI. Verbal informed consent was obtained during PCI procedure in the presence of an independent witness. After the procedure, patients were transferred to the coronary care unit, where they were randomly assigned in a 1:1 ratio to a 4-month oral treatment with either metformin hydrochloride (500 mg twice daily) or a visually matching placebo using block randomization of six patients. Time of administration after successful PCI ranged from 81 to 133 min in the metformin group and 78–134 min in the control group. Written informed consent was obtained during the admission at the coronary care unit from all but one (randomized to placebo). This patient was previously excluded from further analysis [6] leaving in total 379 patients in this 2-year follow-up study. All patients were treated concomitantly according to the European practice guidelines for a STEMI [1]. Follow-up visits were performed by investigators blinded to treatment allocation at one and 2 years after randomization. During follow-up, NT pro-BNP levels were measured at baseline, and on average 3 h, 12 h, 2 weeks, 6–8 weeks, 4 months and 1 year after baseline. During these visits, physical examination, clinical assessment, and 12-lead electrocardiography were performed and standard laboratory assessment was repeated at 1 year.

The principal secondary clinical outcome parameter of the current study was the combined incidence of MACE (defined as cardiovascular death, recurrent MI or target lesion revascularization) 2 years after randomization. During the time of follow-up, all predefined clinical endpoints (including death, reinfarction, recurrent coronary intervention, stroke, hospitalization for heart failure or chest pain, implantable cardioverter defibrillator (ICD) implantation, and new-onset DM [defined as either receiving antidiabetic medication or a glycated hemoglobin (HbA1c) level of ≥6.5% or a glucose level (≥11.1 mmol/L) compatible with this diagnosis] were also assessed and adjudicated by an independent, blinded to allocation, adjudication committee [7, 9]. Additional secondary efficacy measures were all-cause mortality, the individual components of MACE, new-onset DM, and NT pro-BNP levels.

Differences between means of continuous variables with a normal distribution were assessed using the two-tailed Student’s *t* test. Log transformation was used to convert not normally distributed data to a normal distribution. Differences in effect measurements and their 95% confidence intervals between the control group and metformin group were presented when indicated. Logistic regression with concomitant odds ratio (OR) was used to test the treatment effect on the endpoint of new-onset DM, as dates were not available. Associations between the treatment groups and the predefined clinical endpoints were analyzed using the Mantel–Cox or log-rank test and presented with hazard ratios (HR). Kaplan–Meier survival curves were used to present all-cause mortality and MACE incidences. Cox proportional hazard regression was performed to adjust for covariates. Differences in medication use between the control and metformin group were evaluated with the Fisher’s exact test. Linear mixed-effect models were used to assess NT pro-BNP levels over time (with last observation carried forward when missing) between treatment groups. A *P* value of <0.05 was considered to indicate statistical significance. Analyses were performed using Stata version 13.0 (StataCorp).

## Results

Baseline characteristics of patients included in the GIPS-III trial were previously reported and are similar between the two treatment groups [6]. In short, 191 patients were included in the metformin group and 188 in the placebo group. The majority was male, Caucasian, were current smokers, and had hypercholesterolemia. A history of hypertension was present in 30% of the patients. The most common infarct-related artery was the right coronary artery with a prevalence of 45 and 68% of patients had single vessel disease. Furthermore, the majority of patients had myocardial blush grade 3 and 91% had TIMI flow 3 post-PCI. Median peak creatine kinase MB was 163 (interquartile range (IQR) 68; 343) U/L in the metformin group and 159 (IQR 69–300) U/L in the placebo group. Data on mortality was available for the entire follow-up period of all 379 patients. In 7 (2%) patients, 4 in the placebo group and 3 in the metformin group, follow-up data on MACE was limited ranging from 129 to 577 days; for all other patients, 2-year follow-up visits were completed.

**Fig. 1:**
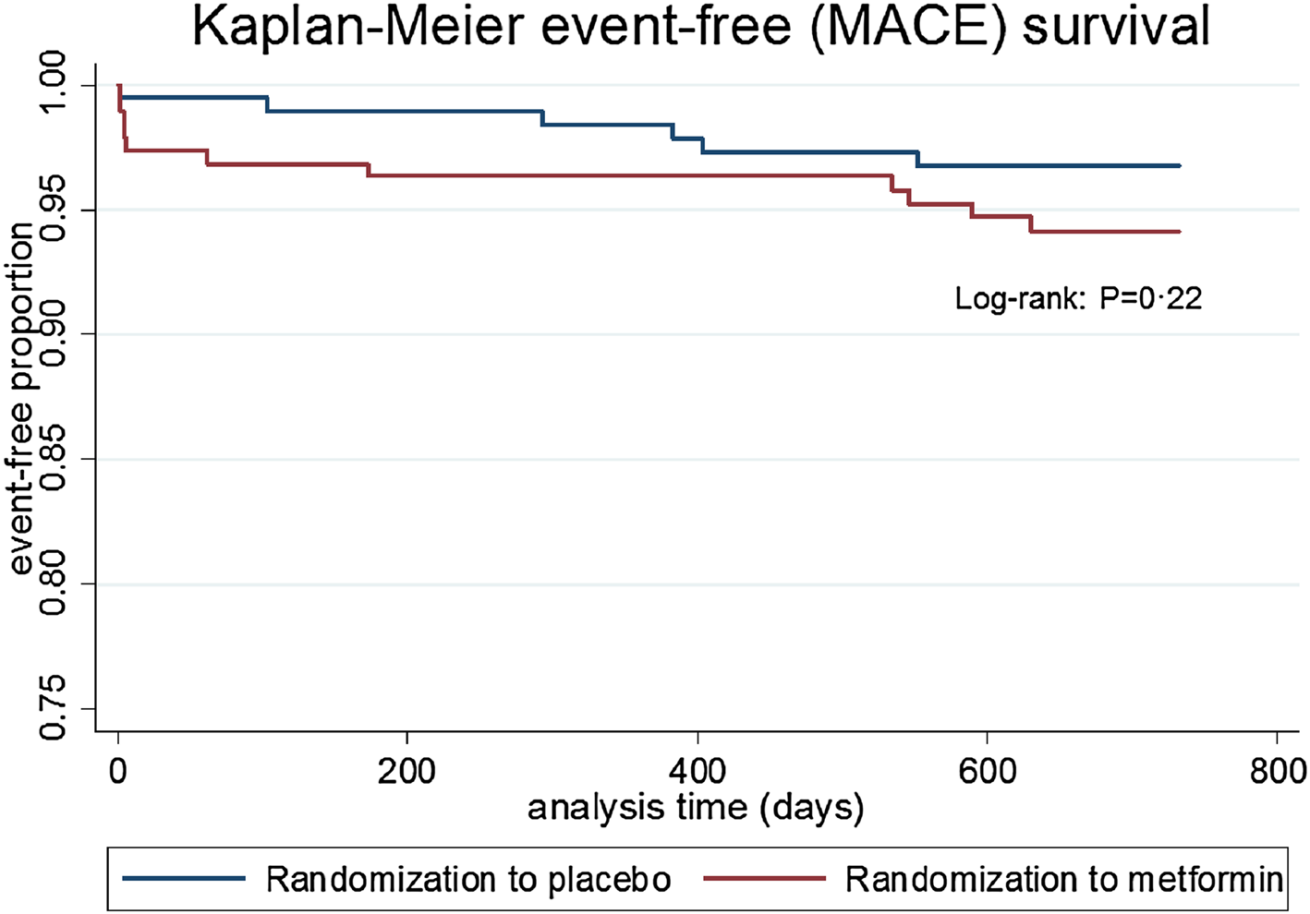
Kaplan–Meier curve representing MACE-free survival during 2-year follow-up in the metformin and placebo treatment groups. MACE-free survival was not significantly different between the groups (Log-rank test *P* = 0.22)

During follow-up, MACE occurred in 17 patients (4.5%) (Table 1). Two MACE, both target lesion revascularizations, occurred at the day of randomization. Four patients died during follow-up, 1 patient due to a cardiovascular cause. Thirteen patients had a recurrent MI of which six patients also underwent a target lesion revascularization. MACE occurred in 11 (5.8%) patients treated with metformin compared to 6 (3.2%) patients treated with placebo (HR 1.84, confidence interval (CI) 0.68–4.97, *P* = 0.22, Fig. 1).

**Table 1:** Secondary endpoints after 2-year follow-up

Secondary endpoint	Total (*N* = 379)	Metformin (*N* = 191)	Placebo (*N* = 188)	*P* value
MACE (%)	17 (4.5)	11 (5.8)	6 (3.2)	0.22
Cardiovascular death (%)	1 (0.3)	1 (0.5)	–	N.A.
Reinfarction (%)	13 (3.4)	8 (4.2)	5 (2.7)	0.41
Target lesion revascularization (%)	9 (2.4)	5 (2.6)	4 (2.1)	0.74
Death (%)	4 (1.1)	3 (1.6)	1 (0.5)	N.A.
Non-cardiovascular death (%)	3 (0.8)	2 (1.1)	1 (0.5)	N.A.
STEMI (%)	5 (1.3)	2 (1.1)	3 (1.6)	0.64
NSTEMI (%)	8 (2.1)	6 (3.1)	2 (1.1)	0.16
Target vessel revascularization (%)	7 (1.9)	2 (1.1)	5 (2.7)	0.25
Non-target lesion revascularization (%)	66 (17.4)	40 (20.9)	26 (13.8)	0.07
CABG (%)	15 (4.0)	10 (5.2)	5 (2.7)	0.20
Hospitalization for heart failure (%)	3 (0.8)	3 (1.6)	–	N.A.
Hospitalization for chest pain (%)	43 (11.4)	23 (12.0)	20 (10.6)	0.60
ICD implantation (%)	13 (3.4)	8 (4.2)	5 (2.7)	0.41
Stroke (%)	3 (0.8)	1 (0.5)	2 (1.1)	N.A.
New-onset diabetes mellitus (%)	66 (17.4)	34 (17.8)	32 (17.0)	0.84

Target lesion revascularization is defined as a percutaneous coronary intervention in the same coronary segment as the culprit lesion of the index event. Target vessel revascularization is defined as a percutaneous coronary intervention in the same culprit vessel, but not the same coronary segment of the index event

*MACE* major adverse cardiac events, *STEMI* ST-segment elevation myocardial infarction, *NSTEMI* non-ST-segment elevation myocardial infarction, *CABG* coronary artery bypass graft, *ICD* implantable cardioverter defibrillator, *N.A.* not applicable

**Fig. 2:**
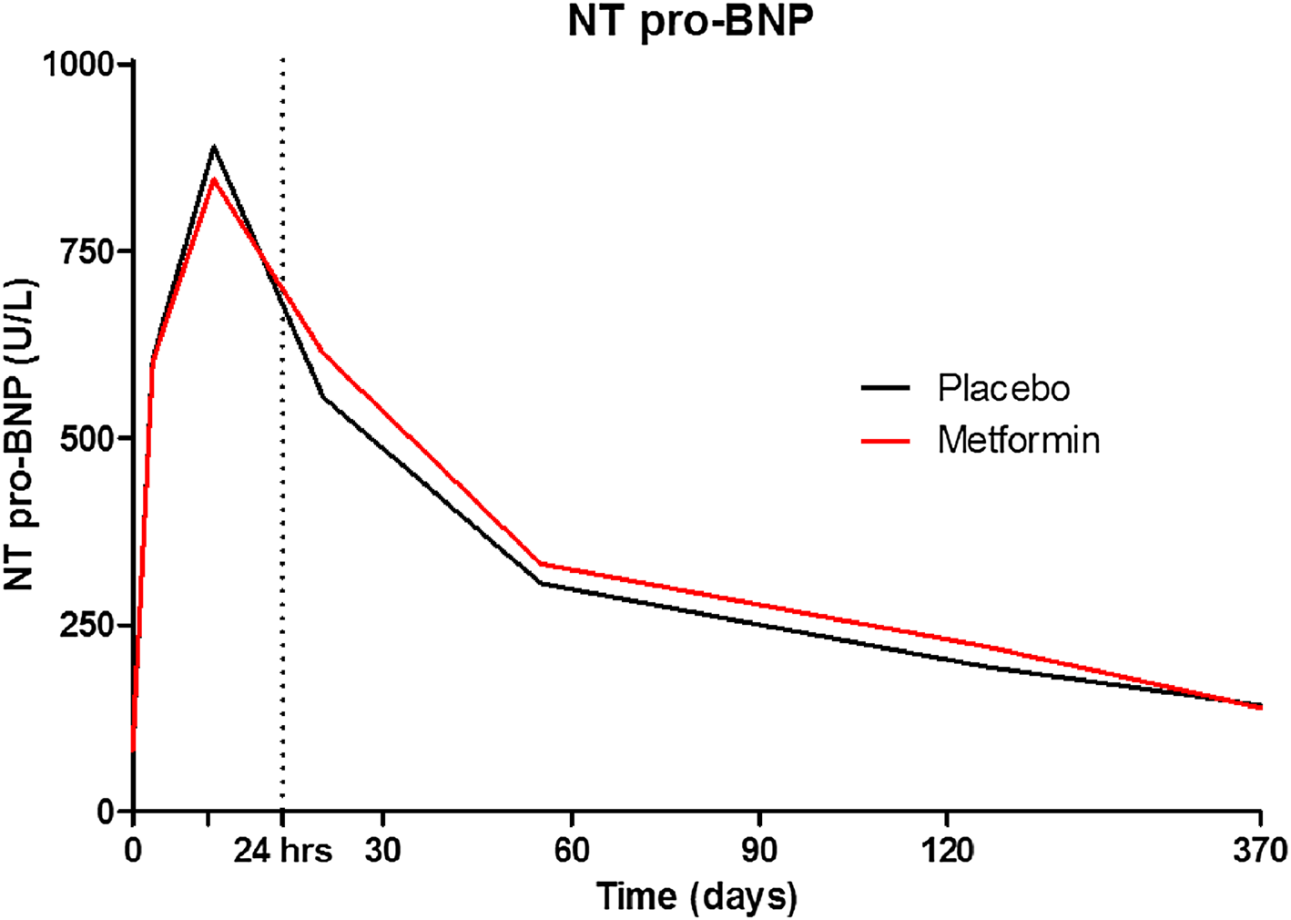
Median NT pro-BNP levels during 2-year follow-up in the metformin treatment and placebo treatment group. Levels were not significantly different between the groups (linear mixed effects *P* = 0.35)

Three patients died of a non-cardiovascular cause. Revascularizations of non-infarct-related artery stenoses visualized during initial STEMI were mainly performed in a staged manner and included in the endpoint as defined by our protocol. Therefore, the incidence of non-target lesion revascularizations was substantial (17.4%). Of all non-target lesion revascularizations, 84.8% (*N* = 56) were identified during PCI of the initial STEMI event. In the other ten patients receiving a non-target lesion revascularization, the median time was after 386 days (range 11–627 days) of follow-up. During follow-up, 66 (17.4%) patients developed DM. The individual components of MACE and other predefined clinical outcome parameters were also not significantly different between metformin and placebo treated patients (Table 1). Three (1.6%) patients in the metformin group died compared to 1 (0.5%) in the placebo group. New-onset DM was 34 (17.8%) in metformin and 32 (17.0%) in placebo treated patients (OR 1.15, CI 0.66–1.98, *P* = 0.84). Potential confounding by differences in concomitant medication use during 2-year follow-up was evaluated and no differences were observed (Table 2). NT pro-BNP levels over time were not significantly different between treatment groups (*P* = 1.00) (Fig. 2).

**Table 2:** Medication use during 2-year follow-up

Medication	Total (*N* = 379)	Metformin (*N* = 191)	Placebo (*N* = 188)	*P* value
Medication use at discharge (%)	379 (100)			
Aspirin (%)	367 (96.8)	184 (96.3)	183 (97.3)	0.77
Thienopyridine (%)	379 (100)	191 (100)	188 (100)	1.00
Coumarin (%)	20 (5.3)	13 (6.8)	7 (3.7)	0.25
Beta blocker (%)	362 (95.5)	179 (93.7)	183 (97.3)	0.14
ACE inhibitor or ARB (%)	301 (79.4)	158 (82.7)	143 (76.1)	0.13
Statin (%)	377 (99.5)	190 (99.5)	187 (99.5)	1.00
Antidiabetic drugs (%)	7 (1.9)	4 (2.1)	3 (1.6)	1.00
Medication use at 3-4 months (%)	356 (93.9)			
Aspirin (%)	346 (97.2)	171 (96.1)	175 (98.3)	0.34
Thienopyridine (%)	349 (98.0)	174 (97.8)	175 (98.3)	1.00
Coumarin (%)	26 (7.3)	17 (9.6)	9 (5.1)	0.15
Beta blocker (%)	340 (95.5)	170 (95.5)	170 (95.5)	1.00
ACE inhibitor or ARB (%)	276 (77.5)	133 (74.7)	143 (80.3)	0.25
Statin (%)	343 (96.4)	173 (97.2)	170 (95.5)	0.57
Antidiabetic drugs (%)	7 (2.0)	5 (2.8)	2 (1.1)	0.45
Medication use at 2 years (%)	370 (97.6)			
Aspirin (%)	356 (96.2)	178 (95.7)	178 (96.7)	0.79
Thienopyridine (%)	24 (6.5)	9 (4.8)	15 (8.2)	0.21
Coumarin (%)	25 (6.8)	15 (8.1)	10 (5.4)	0.41
Beta blocker (%)	316 (85.4)	160 (86.0)	156 (84.8)	0.77
ACE inhibitor or ARB (%)	249 (67.3)	120 (64.5)	129 (70.1)	0.27
Statin (%)	332 (89.7)	172 (92.5)	160 (87.0)	0.09
Antidiabetic drugs (%)	23 (6.2)	11 (5.9)	12 (6.5)	0.83

*ACE* angiotensin converting enzyme, *ARB* angiotensin receptor blocker

## Discussion

The GIPS-III trial is the first prospective study evaluating the effect of 4 months of metformin treatment in patients without DM, presenting with STEMI. The primary endpoint of the GIPS-III trial, LVEF at 4 months, was not affected by metformin treatment [6]. In the present study we now provide 2-year follow-up data and evaluated predefined secondary endpoints including MACE. We observed a similar incidence of MACE between patients who received metformin or placebo treatment for 4 months after STEMI. Other predefined secondary endpoints including all-cause mortality and new-onset DM did not differ between the treatment groups.

Previous data on the effects of metformin in MI originate predominantly from animal experimental or human observational data and are inconsistent. For example, some studies have reported a decrease in myocardial infarct size due to metformin [4] while others suggested no effect [10]. It has also been suggested that in patients with DM presenting with MI, chronic metformin use might reduce 30-day all-cause mortality, although 12-month all-cause mortality was not significantly different [11]. Metformin also did not affect LVEF in these patients at 12 months [11]. The prospective Metformin in Coronary Artery Bypass Graft (MetCAB) trial investigated pretreatment of metformin in 100 patients undergoing CABG, and did not observe a reduction in periprocedural myocardial injury based on assessment of Troponin I levels [12]. Though, periprocedural myocardial injury is mostly limited and not comparable to the extent of myocardial injury caused by MI. Besides, a different underlying pathophysiologic process might play a role, which could be an explanation why metformin was not effective.

In the current analyses of GIPS-III we did not observe a beneficial effect of 4 months of metformin treatment on long-term clinical outcomes, even when taking into account potential confounding by concomitant medical therapy. The incidence of MACE during 2-year follow-up was low and the same applies for heart failure hospitalizations and ICD implantations. This is probably due to the efficient local STEMI protocol resulting in short ischemia times and successful reperfusion in the majority of patients. In addition, all STEMI patients received medical therapy as recommended by current guidelines [1]. Patients of the GIPS-III trial had relatively small infarct size and largely preserved LVEF as measured by CMR at 4 months [6], both of which are associated with favorable outcomes [13, 14]. However, it should be mentioned that 28% of the study population did not undergo CMR and selection bias may have distorted these findings. One study showed that CMR dropouts in general had a worse baseline clinical risk profile, although this had no effect on clinical endpoints [15]. Apart from the expected non-target vessel revascularizations diagnosed during the initial STEMI event, the incidence of revascularizations in the GIPS-III trial was remarkably low. As suggested in the review of Lexis et al. [16], metformin could play a role in the prevention of restenoses, although this was not confirmed in our study. Limitations of this work that warrant consideration are the fact that the GIPS-III trial was primarily designed to detect the effect of metformin on LVEF with 80% power. Besides, other long-term follow-up studies with smaller patient populations found MACE incidences ranging from 25 to 35% as compared to 4.5% in the GIPS-III cohort [17–19]. The unexpected low incidence of MACE, which might have ensued from the efficient local STEMI protocol resulting in relatively small infarct size, has led to insufficient power to rule out beneficial effects of metformin on predefined clinical endpoints with certainty. The overall low rate of new cardiovascular events implies that with the current treatment strategy of reperfusion therapy and secondary prevention we are able to accomplish a favorable outcome in most patients. Hence, we might have reached a phase in clinical care to which additional therapies might be of only limited additional value when STEMI patients receive optimal reperfusion therapy.

Several considerations have been discussed previously in defense of the moderate dose of metformin used in the GIPS-III trial [6]. In an open-label randomized controlled clinical trial including patients with metabolic syndrome undergoing elective PCI, treatment with a total dose of 750 mg metformin prior to the procedure resulted in less cardiac biomarker release and a favorable outcome at 1-year follow-up [20]. As the lower dose used in this study with a similar population already showed to be effective, a higher dose up to 3000 mg is not expected to give a different outcome in our study.

The timing and duration of metformin treatment might play a crucial role in its potential cardioprotective effects. The majority of the previous experimental and observational data reported protective effects in the setting of metformin administration before or during reperfusion [4]. In the GIPS-III trial, metformin was administered directly after PCI and effective plasma levels were probably achieved hours later, resulting in a shorter window of opportunity to modify ischemia–reperfusion injury. In animal models, ischemic reperfusion injury has been suggested to contribute up to 50% to the final size of MI [21]. We cannot exclude that metformin therapy initiated prior to PCI might indeed reduce myocardial infarct size as has been suggested by prior data [4, 22, 23]. Several mechanisms have been postulated to play a key role in explaining the effect of early treatment [24–27]. Nonetheless, we did not observe a positive effect with the applied strategy of post-PCI metformin administration on infarct size nor on other endpoints.

Furthermore, sex-dependent differences in the metabolic and functional response to metformin have been suggested [10]. Metformin therapy decreases fatty acid clearance which consequently results in increased fatty acid plasma levels and myocardial fatty acid utilization and oxidation in men, which has been linked to adverse clinical outcomes in myocardial ischemia setting [28]. The opposite has been observed in women. In the GIPS-III trial the majority of patients were males, which limits us to detect a potential positive effect of metformin treatment in females.

Previous studies demonstrated that metformin therapy can prevent or delay the onset of type II DM [29, 30]. We did not observe an effect of 4 months metformin treatment on the incidence of DM after 4-month follow-up [7], or in this analysis after 2-year follow-up. The treatment duration of 4 months in the GIPS-III trial, as opposed to 1.5–3 years in other studies, might not be long enough [30, 31], and the pharmacological effects of metformin might not persist after discontinuation. With 17% of patients developing DM our study does confirm that patients, after their first STEMI, should be followed for new-onset DM. Starting with lifestyle interventions, especially in case of impaired glucose tolerance, is recommended in STEMI patients [1].

Currently, several clinical trials, in ischemic as well as non-ischemic settings, are evaluating the effect of metformin on diverse cardiovascular endpoints (Online Resource 1). These trials will provide further insight into the potential clinical value of metformin treatment in cardiovascular disease.

To conclude, in this 2-year follow-up of the GIPS-III trial we observed no differences between STEMI patients treated with metformin versus placebo on predefined secondary endpoints, including MACE. Moreover, no effect was seen on the incidence of new-onset DM in both groups. On the one hand, the overall low incidence of MACE prohibited us to definitely rule out long-term beneficial effects of metformin in STEMI patients without DM. On the other hand, the low rate of new cardiovascular events in a population with optimal reperfusion therapy and secondary prevention might imply that we have reached a phase in clinical care to which additional therapies are of only limited additional value. This might also be a consideration in the design of future interventional studies in STEMI patients.

## Electronic supplementary material

Below is the link to the electronic supplementary material.

Supplementary material 1 (DOCX 18 kb)
