# Supplementary material for: Two-year follow-up of 4 months metformin treatment vs. placebo in ST-elevation myocardial infarction: data from the GIPS-III RCT
Source: Clin Res Cardiol. 2017 Jul 28;106(12):939–46. doi: 10.1007/s00392-017-1140-z (PMC5696505; doi:10.1007/s00392-017-1140-z)
Supplement: Supplementary file 1 — Supplementary material 1 (DOCX 18 kb) [file 392_2017_1140_MOESM1_ESM.docx]

Clinical Research in Cardiology

**Two-year follow-up results of four months metformin treatment vs. placebo in STEMI: the GIPS-III RCT**

Minke H.T. Hartman, M.D., Jake K.B. Prins, BSc., Remco A.J. Schurer, M.D., Erik Lipsic M.D., Ph.D., Chris P.H. Lexis M.D., Ph.D., Anouk N.A. van der Horst-Schrivers, M.D., Ph.D., Dirk J. van Veldhuisen, M.D., Ph.D., Iwan C.C. van der Horst, M.D., Ph.D., Pim van der Harst, M.D., Ph.D.

Corresponding author: Prof. Dr. P. van der Harst, University of Groningen, University Medical Center Groningen, Department of Cardiology, the Netherlands, Hanzeplein 1, 9700 RB Groningen. Telephone number: +31 5036 12355. Fax number: +31 50 36 14884. E-mail: [p.van.der.harst@umcg.nl](mailto:p.van.der.harst@umcg.nl).

**Supplementary Table 1. Summary of ongoing clinical trials**

| **Author, country,**  **Trial registration** | **Status,**  **N** | **Patient group** | **Intervention & primary endpoint** |
| --- | --- | --- | --- |
| Xiang Guang-da, China[1], NCT01879293 | Completed,  N*=*120 | non-diabetic patients with IHD and LVH | Effect of metformin 1500mg/day vs. placebo for 1 year on change in LVM as measured with CMR |
| Chim Lang, UK[2], 25545400/ NCT02226510 | Active, not recruiting, N*=*64 | non-diabetic patients with IHD and LVH | Effect of metformin 1000-2000mg/day vs. placebo for 1 year on change in LVM index as measured with CMR |
| Terezie Pelikanova, Czech Republic[3], NCT01690091 | Recruiting,  N*=*40 | type 2 diabetes mellitus patients with HF | Effect of metformin 500-1000mg/day vs. placebo for 3 months on insulin sensitivity |
| Ricardo Ladeiras-Lopes, Portugal[4], NCT02017561 | Recruiting,  N*=*54 | non-diabetic patients with metabolic syndrome and LV diastolic dysfunction | Effect of metformin 500-1000mg/day vs. lifestyle counseling for 2 years on change in mean early diastolic mitral annular velocity as assessed by tissue Doppler echocardiography |
| Simon Griffin, United Kingdom[5],  ISRCTN34875079 | Ongoing,  N*=*11,834 | non-diabetic hyperglycemia patients | Effect of metformin 1500mg/day vs. placebo in preventing cardiovascular events over 5 years |

Abbreviations: CMR, cardiac magnetic resonance imaging; HF, heart failure; IHD, ischemic heart disease; LVH, left ventricular hypertrophy; LV, left ventricular; LVM, left ventricular mass; mg, milligram; UK, United Kingdom of Great Britain.

**Supplementary References**

1. Guang-da X (2015) Metformin Reduces Left Ventricular Mass in Patients With Ischemic Heart Disease. In: clinicaltrials.gov. <https://clinicaltrials.gov/ct2/show/NCT01879293>. Accessed April 3, 2017.

2. Lang CC (2016) MetfoRmin and Its Effects on Left Ventricular Hypertrophy in Normotensive Patients With Coronary Artery Disease (MET-REMODEL). In: clinicaltrials.gov. <https://clinicaltrials.gov/ct2/show/NCT02226510>. Accessed April 3, 2017.

3. Pelikanova T (2016) Cardioprotective and Metabolic Effects of Metformin in Patients With Heart Failure and Diabetes (CARMET). In: clinicaltrials.gov. <https://clinicaltrials.gov/ct2/show/NCT01690091>. Accessed April 3, 2017.

4. Ladeiras-Lopes R (2016) Metformin in the Diastolic Dysfunction of Metabolic Syndrome (MET-DIME). In: clinicaltrials.gov. <https://clinicaltrials.gov/ct2/show/NCT02017561>. Accessed April 3, 2017.

5. Griffin S (2016) The Glucose Lowering In Non-diabetic hyperglycaemia Trial (GLINT) - Glucose lowering in those at risk of diabetes. In: ISRCTN Registry. <http://www.isrctn.com/ISRCTN34875079>. Accessed April 3, 2017.
